# Staphylococcal Complement Evasion Protein Sbi Stabilises C3d Dimers by Inducing an N-Terminal Helix Swap

**DOI:** 10.3389/fimmu.2022.892234

**Published:** 2022-05-25

**Authors:** Rhys W. Dunphy, Ayla A. Wahid, Catherine R. Back, Rebecca L. Martin, Andrew G. Watts, Charlotte A. Dodson, Susan J. Crennell, Jean M. H. van den Elsen

**Affiliations:** ^1^ Department of Biology and Biochemistry, University of Bath, Bath, United Kingdom; ^2^ Department of Pharmacy and Pharmacology, University of Bath, Bath, United Kingdom; ^3^ Centre for Therapeutic Innovation, University of Bath, Bath, United Kingdom

**Keywords:** complement, structural biology, *Staphylococcus aureus*, C3d, 3D domain swapping

## Abstract

*Staphylococcus aureus* is an opportunistic pathogen that is able to thwart an effective host immune response by producing a range of immune evasion molecules, including *S. aureus* binder of IgG (Sbi) which interacts directly with the central complement component C3, its fragments and associated regulators. Recently we reported the first structure of a disulfide-linked human C3d^17C^ dimer and highlighted its potential role in modulating B-cell activation. Here we present an X-ray crystal structure of a disulfide-linked human C3d^17C^ dimer, which undergoes a structurally stabilising N-terminal 3D domain swap when in complex with Sbi. These structural studies, in combination with circular dichroism and fluorescence spectroscopic analyses, reveal the mechanism underpinning this unique helix swap event and could explain the origins of a previously discovered N-terminally truncated C3dg dimer isolated from rat serum. Overall, our study unveils a novel staphylococcal complement evasion mechanism which enables the pathogen to harness the ability of dimeric C3d to modulate B-cell activation.

## Introduction

The central complement component C3 is cleaved into its constituent fragments C3a and C3b by C3 convertase complexes generated by the classical/lectin (C4bC2a) or alternative (C3bBb) pathways, which subsequently trigger effective immune defence mechanisms against microbial pathogens (1). Upon activation of C3, opsonisation of microbial surfaces or cell debris in the surrounding environment by C3b enables clearance *via* opsonophagocytosis, whereas the C3a anaphylatoxin stimulates the inflammatory immune response (1). Our understanding of the mechanisms driving the cleavage of C3 have been advanced greatly by structural studies of native C3 and its fragments C3b and C3c (2, 3). Structures of C3b in complex with factor I (FI) and the short consensus repeat (SCR) domains 1-4 of cofactor factor H have also helped us understand the processes by which C3b is proteolytically cleaved into the potent opsonin fragments iC3b and C3d(g) (4). Additionally, complexes of C3b with FH SCR domains 19 and 20 have been pivotal in explaining the regulatory measures, involving competitive binding to glycosaminoglycans and sialic acids on the host surface, that are in place to prevent the indiscriminate attachment of C3b and its fragments to self surfaces as opposed to non-self surfaces (5, 6).

Remarkably, during C3 activation only 10% of the C3b generated is deposited onto reactive surfaces, while the remaining 90% remains in the fluid phase where the highly reactive Cys-Gln thioester moiety within the thioester-containing C3d domain (TED) of C3 is exposed and undergoes hydrolysis resulting in the formation of C3(H_2_O) (7). In this form, the cysteine free sulfhydryl is freely available and therefore capable of forming dimers of C3b, iC3b and C3d(g), which have been observed in several studies including in serum after ‘aging’ (8) or under non-reducing conditions such as the absence of *N*-ethylmaleimide (9, 10). Thioester linked C3b dimers have been reported to act as potent alternative pathway (AP) activators when complexed with IgG (11), enable the formation of AP C5 convertases by serving as a binding platform for factor B fragment Bb (12, 13), promote the release of histaminase from human polymorphonuclear leukocytes (8) and bind to complement receptor 1 (CR1) with a 25-fold higher affinity than monomeric C3b (9). More recently, we have reported on a crystal structure of a cysteine C17 disulfide-linked human C3d dimer and revealed that this dimeric C3d can bind to and crosslink CR2, enabling the modulation of B cell activation, which could potentially affect immunotolerance (14).

*Staphylococcus aureus* evasion proteins have been shown to effectively interfere with the process of complement activation. Along with elucidating their role in thwarting the immune system, structural and functional studies of these factors have been instrumental in furthering our understanding of the molecular mechanisms controlling C3 activation (15). Of the C3-interacting staphylococcal complement evasion proteins (16–21), Sbi is of particular interest as it is able to activate C3 through the alternative complement pathway and thereby cause fluid phase consumption of C3 (22–24). In addition to two N-terminal domains that bind the Fc region of IgG in a fashion similar to that of protein A (domains I and II) (25), we have previously shown that Sbi domains III and IV interact with complement proteins, whereby Sbi-IV binds to C3 fragments by interacting with the acidic residue-lined concave face of monomeric C3d (16), and in combination with Sbi domain III, activates the alternative complement pathway. We have recently shown that this property of Sbi can be harnessed as a vaccine adjuvant that promotes the opsonisation of antigens with a ‘natural’ coat of C3 breakdown products (26) *via* AP activation-mediated consumptive cleavage of C3 (22).

In this study we present the structure of a C3d^17C^ dimer in complex with *S. aureus* immune evasion protein Sbi and show that through this interaction the dimer undergoes a unique N-terminal 3D domain swap. In combination with spectroscopic techniques such as circular dichroism and fluorescence spectroscopy we delineate the potential mechanism by which this structurally stabilising domain swap occurs. Our findings propose a potential new immune evasion mechanism enabling *S. aureus* to highjack the immunomodulatory properties of disulfide linked C3d dimers.

## Results

### Sbi-IV Induces a Unique 3D Domain Swap in Dimeric C3d

**Figure 1:**
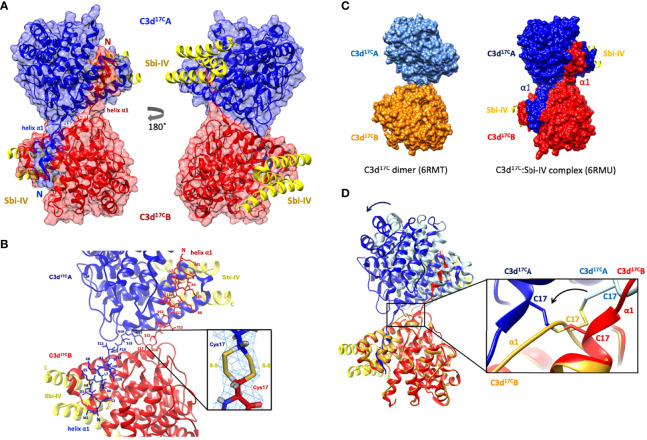
Crystal structure of a human C3d^17C^ dimer in complex with Sbi-IV at 2.4 Å resolution. **(A)** Ribbon diagram of the C3d^17C^:Sbi-IV dimer complex showing the cysteine C17 disulfide linked monomers in red and blue, respectively. A reciprocal 3D domain swap of both N-terminal α1 helices can be observed between the two C3d chains in the dimer, involving amino acids M1-G16. **(B)** Enlarged view of the C3d^17C^ dimer interface showing the side chains of helix α1 residues M1-C17. Inset: 2Fo-Fc electron density contoured at 1.0 σ of two possible interchain disulfide bonds (conformer A: 2.04 Å, conformer B: 2.06 Å) with a preference for the conformer B disulfide (occupancy: 0.66). The structure of C3d^17C^:Sbi-IV dimer complex was submitted to the Protein Data Bank with PDB accession code: 6RMU. See **Supplementary Tables S1, S2** for data collection and refinement statistics. **(C)** Comparison of the unliganded C3d^17C^ dimer (PDB accession code 6RMT, with the molecular surface representations of the C3d monomers highlighted in gold and light blue) and the C3d^17C^:Sbi-IV dimer complex (6RMU, with the C3d monomers shown in red and dark blue, and Sbi-IV in yellow), clearly showing the 3D domain swap of the N-terminal α1 helices in the latter dimer. **(D)** Superpositioning of the C3d A chains in both unliganded and Sbi-IV bound C3d dimer structures, highlighting the overall changes in relative orientation of the C3d B-chain within the unliganded C3d^17C^ and helix-swapped dimers upon superpositioning of A-chain (left), and the conformational differences in disulfide bond arrangement between both dimers (right). The images produced in this figure were generated using Chimera (27).

Building on our recent structural and functional findings about the C3d^17C^ dimer (14), we set out to determine the structure of a complex between dimeric C3d^17C^ and Sbi-IV. The C3d^17C^:Sbi-IV dimer complex (**Figure 1**, data collection and refinement statistics can be found in **Supplementary Tables S1, S2**) shows two molecules of Sbi-IV binding to the opposing concave binding surfaces on the C3d^17C^ dimer (**Figure 1A**) *via* interactions identical to those observed in the previously reported C3d^17A^:Sbi-IV structure (16). An additional binding mode observed in the C3d^17A^:Sbi-IV structure, with Sbi-IV interacting with the convex surface of C3d close to the thioester region (**Supplementary Figure S2**), is absent in the C3d^17C^:Sbi-IV dimer complex, prevented by the tight dimer interactions. Intriguingly, and in clear contrast to the unliganded C3d^17C^ dimer, the Sbi-IV bound C3d^17C^ dimer has undergone a 3D domain swap, whereby both the N-terminal α1 helices have been reciprocally exchanged between the C3d^17C^ molecules within the dimer (**Figure 1A**). As detailed in **Figure 1B**, the helix α1 region M1 – G16 (residue numbering based on the crystal structure), located N-terminally from C17, has undergone a full polypeptide chain exchange in both C3d^17C^ molecules, recreating identical interactions with the opposing C3d recipient without causing appreciable differences in relative orientation when compared to both monomers in the unliganded C3d^17C^ dimer (PDB accession code 6RMT; main-chain RMSDs: 0.55 Å, residues 20-290 in Chain A; 0.15 Å, residues 20-290 in chain B). 2Fo-Fc electron density maps for the critical loop regions of the swapped α1 helices for each chain are shown in **Supplementary Figure S1**). This unique structural 3D domain swap phenomenon is highlighted in **Figure 1C**, showing a comparison between the unliganded C3d^17C^ dimer and the C3d^17C^:Sbi-IV dimer complex. Superpositioning of the A chains in the unliganded C3d^17C^ dimer and the C3d^17C^:Sbi-IV dimer complex reveals a slight but significant change in overall relative orientation of both monomers in the C3d^17C^:Sbi-IV dimer and highlights the resulting change in conformation of the dimer disulfide bond as a result of the N-terminal helix swap (**Figure 1D**).

### Helix Swapping Structurally Stabilises Dimeric C3d

In addition to stabilising the C17-C17 interchain disulfide, the helix α1 swap causes a substantial increase (640%) in joint buried surface area from 698 Å^2^ in the unliganded C3d^17C^ dimer to 4474 Å^2^ in the Sbi-IV-bound helix-swapped C3d^17C^ dimer. This expansion in interface area is supported by 13 additional hydrogen bonds and 4 ionic interactions which are absent in the unliganded dimer (**Supplementary Figure S3**).

### The Role of the Convex C3d Binding Mode of Sbi-IV in the Helix Swap

**Figure 2:**
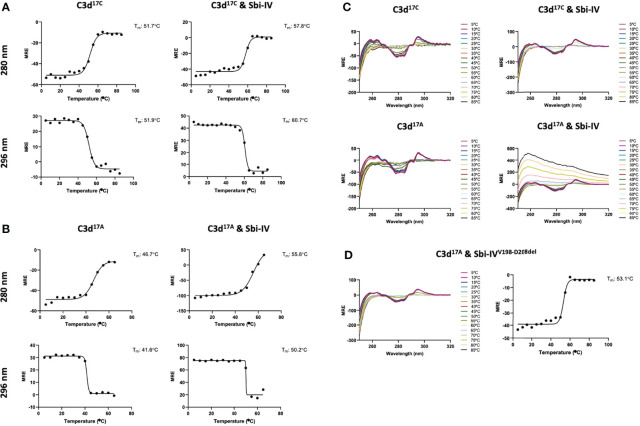
Near-UV thermal melt circular dichroism spectroscopic studies of C3d^17C^ in the absence or presence of wild-type Sbi-IV. **(A)** Melting temperature (T_m_) calculations for C3d^17C^ in the absence (Left) and presence (Right) of Sbi-IV, illustrated as Boltzmann sigmoidal fits of thermal denaturation curves at 280 nm (Top) and 296 nm (Bottom), showing an increase in T_m_ of 6°C and 9°C at 280 and 296 nm respectively when Sbi-IV is present. **(B)** Melting temperature (T_m_) calculations for C3d^17A^ in the absence (Left) and presence (Right) of Sbi-IV, illustrated as Boltzmann sigmoidal fits of thermal denaturation curves at 280 and 296 nm, this shows an increase in T_m_ of ~9°C and at 280 and 296 nm respectively when Sbi-IV is present. MRE: mean residue ellipticity (deg cm^2^ dmol^-1^ residue^-1^). **(C)** CD spectra showing that C3d^17A^ in the presence of wild-type Sbi-IV undergoes aggregation and precipitation at high temperatures **(D)**. Precipitation of C3d^17A^ is not observed in the presence of a truncated Sbi-IV mutant (Sbi-IV^V198-D208del^), lacking the N-terminal region of Sbi-IV helix α1, and Sbi-IV single mutant S199A. A T_m_ increase of 6.5°C indicates that the truncated Sbi-IV^V198-D208del^ mutant hasn’t lost its ability to bind C3d^17A^.

To further investigate the potential role of the interaction between Sbi-IV and helix α1 on the concave face of C3d (**Supplementary Figure S2**) we employed near-UV (320-250 nm) circular dichroism (CD) spectroscopic analyses of C3d^17C^ and C3d^17A^ collected as a function of temperature, where the absorption of aromatic side chains and disulfide bonds, absent in Sbi-IV, provides information about changes in C3d’s tertiary structure (28) and binding energy of complex formation with Sbi-IV (**Figure 2**). After analysis of the sigmoidal fits of thermal denaturation curves, Sbi-IV was found to increase the melting temperature (ΔT_m_) of C3d^17C^ by 7.4°C from 51.8 ± 0.1°C in the absence of Sbi-IV to 59.2 ± 1.5°C in the presence of Sbi-IV (**Figure 2A**; averaged ΔT_m_ at 280 and 296 nm**)**, reflecting the binding energy of the complex. In comparison, the presence of Sbi-IV raises the ΔT_m_ of C3d^17A^ by 9°C, indicating a slightly stronger interaction energy for the binding between Sbi-IV and monomeric C3d^17A^ (**Figure 2B**; averaged DT_m_ at 280 and 296 nm).

Interestingly, whilst monomeric C3d^17A^ is thermally stable, it becomes unstable in the presence of wild-type Sbi-IV and unlike dimeric C3d^17C^ undergoes precipitation at temperatures above 65°C (**Figure 2C**). Intriguingly, the precipitation of C3d^17A^ does not occur in the presence of an N-terminally truncated Sbi-IV mutant (V198-D208del; residue numbering based on UniProt: Q2FVK5) lacking key residues that were previously shown to interact with helix α1 and its linker on the convex surface of C3d by crystal structure and NMR analyses of the C3d^17A^:Sbi-IV complex (16) (**Supplementary Figure S2**). Whilst the ~7°C increase in T_m_ (**Figure 2D**) indicates that Sbi-IV^V198-D208del^ is still able to bind C3d^17A^, the lack of a temperature-induced precipitation in the presence of Sbi-IV^V198-D208del^, suggests a potential role for the convex C3d binding mode of Sbi-IV in the displacement of the N-terminal α1 helix of C3d, whereby binding of Sbi-IV destabilises the helix, leading to aggregation and precipitation

**Figure 3:**
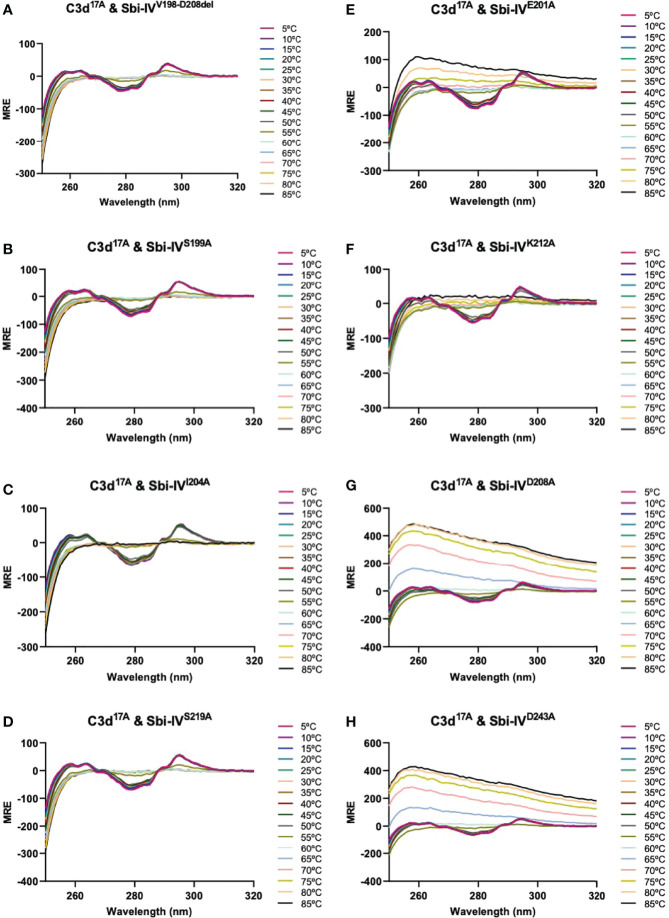
Comparison of near-UV thermal melt circular dichroism spectroscopic studies of C3d^17A^ in the absence or presence of a truncated Sbi-IV mutant or Sbi-IV single mutants. **(A)** Whilst C3d^17A^ in the presence of wild-type Sbi-IV undergoes aggregation and precipitation at high temperatures (**Figure 2C**), this is not observed in the presence of truncated mutant Sbi-IV^V198-D208del^, lacking the N-terminal region of Sbi-IV helix α1, and Sbi-IV single mutants S199A **(B)**, I204A **(C)** and S219 **(D)**. A reduced level of C3d^17A^ aggregation is observed in the presence of Sbi-IV single mutants E201A **(E)** and K212A **(F)**, whilst Sbi-IV single mutants D208A **(G)** and D243A **(H)** show aggregation levels comparable to those caused wild-type Sbi-IV.

We further explored the potential role of Sbi-IV’s convex C3d binding mode in the helix swap observed in the C3d^17C^:Sbi-IV dimer complex by examining single amino acid substitutions within Sbi-IV, which were chosen based on the Sbi-IV residues identified in the convex C3d binding mode of Sbi-IV, observed in the crystal structure of the C3d^17A^:Sbi-IV complex and confirmed by subsequent NMR analyses (16) (**Supplementary Figure S2**). The Sbi-IV mutants were evaluated using near-UV CD analyses in the presence of monomeric C3d^17A^. Similar to the N-terminally truncated Sbi-IV mutant (Sbi-IV^V198-D208del^), alanine substitutions at positions S199, I204 and S219 in Sbi-IV exhibited no observable precipitation or aggregation (**Figures 3A-D**), suggesting that these residues could be important for Sbi-IV’s convex C3d binding mode. E201A and K212A show a reduction of the levels of precipitation (**Figures 3E, F**), indicating that these residues are important for convex surface binding but likely enhance the binding stability once the protein is initially in position. In contrast, substitutions at D208A and D243A exhibited comparable levels of precipitation and aggregation to wild-type Sbi-IV (**Figures 3G, H**) and therefore presumably have less significant roles in the convex surface binding mode.

### Both Sbi-IV and Sbi-III-IV Induce N-Terminal Helix Swapping in C3d

**Figure 4:**
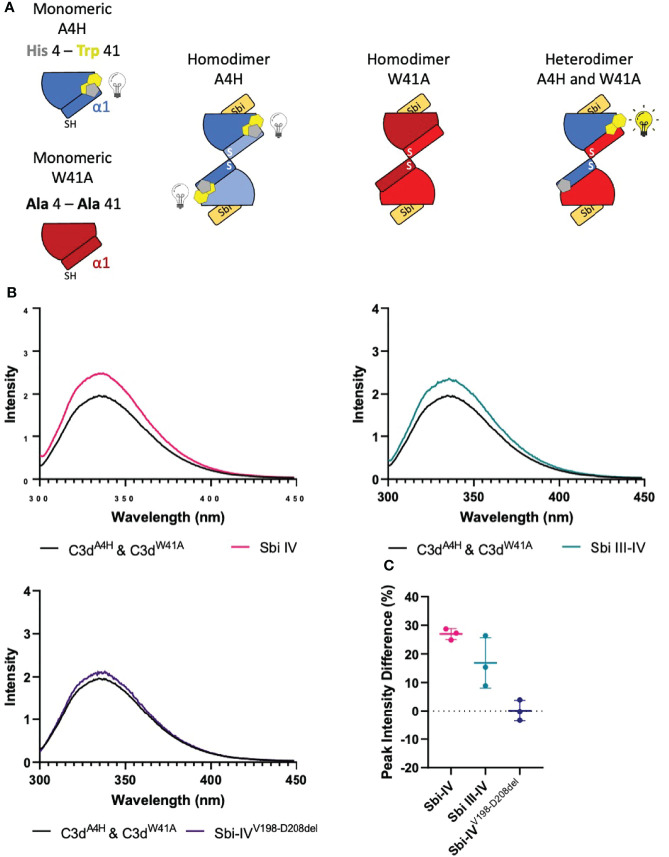
Tryptophan fluorescence spectroscopic studies with C3d^A4H^ and C3d^W41A^ heterodimers in complex with wild-type Sbi-IV, a truncated Sbi-IV mutant and wild-type Sbi-III-IV. Shown is a schematic depicting the C3d^17C^ mutants C3d^A4H^ and C3d^W41A^ and highlighting the natural tryptophan W41 (yellow) and histidine H4 (grey) that was introduced to quench W41’s fluorescence. Homodimers of C3d^A4H^, C3d^W41A^ are expected to show no change of tryptophan fluorescence in the event of an N-terminal helix swap, whilst heterodimers of C3d^A4H^ with C3d^W41A^ should display an increase in tryptophan fluorescence as H4 no longer quenches W41 when the helix swap occurs **(A)**. Fluorescence spectra of C3d heterodimers in the absence and presence of wild-type Sbi-IV, wild-type Sbi-III-IV and truncated Sbi-IV^V198-D208del^ mutant **(B)**. Scatter plot showing the tryptophan fluorescence intensity change with individual readings as well as the mean and SD values, with an average increase of 27%, 17% and 2.5% in the presence of Sbi-IV, Sbi III-IV and truncated Sbi-IV^V198-D208del^ mutant respectively **(C)**.

To investigate whether the helix swap observed in C3d^17C^ dimers is unique to Sbi-IV, we also included Sbi-III-IV in our analyses. Because the aromatic amino acids present in Sbi-III-IV make this construct unsuitable for the near-UV CD temperature melt analyses we used for Sbi-IV, we developed a bespoke tryptophan fluorescence spectroscopy method that exploits the fluorescence quenching effect on tryptophan brought about by histidine residues positioned in close proximity (29). Several C3d^17C^ mutants were generated to either substitute a natural tryptophan (W41, located on a loop between helices α2 and α3) with an alanine or introduce a histidine at position 4 close to W41. As detailed in the **Figure 4A** schematic, solutions of C3d^A4H^ or C3d^W41A^ are expected to form homodimers and show no change of tryptophan fluorescence in the event of an N-terminal helix swap, whilst mixed solutions of C3d^A4H^ with C3d^W41A^ can form heterodimers that should display an increase in tryptophan fluorescence as H4 no longer quenches W41 when the helix swap occurs. As expected, solutions of either C3d^A4H^ or C3d^W41A^ containing homodimers showed slight changes in fluorescence in the presence of Sbi-IV^V198-D208del^, Sbi-IV or Sbi-III-IV (**Supplementary Figure S4**). However, adding these Sbi constructs to mixed solutions of C3d^A4H^ and C3d^W41A^, expected to contain approximately 50% heterodimers of the two mutants, showed various changes in fluorescence. In the presence of Sbi-IV or Sbi-III-IV (**Figures 4B, C**) a clear increase in tryptophan fluorescence (27% and 17%, respectively) was observed when compared to their respective homodimers. These results indicate that W41 in C3d^A4H^ is no longer being quenched by its structural neighbour H4, which is suggestive of the occurrence of a Sbi-induced helix swap event. This is further supported by the reduced change in fluorescence intensity (2.5%) of the C3d^A4H^-C3d^W41A^ heterodimer observed in the presence of Sbi^V198-D208del^ (**Figures 4B, C**), emphasising that W41 remains quenched by H4 as the α1 helices cannot exchange when Sbi is unable to interact with the convex surface of C3d.

## Discussion

We recently we presented the first crystal structure of dimeric human C3d^17C^ and uncovered its potential role in modulating B cell activation (14). In this paper we discover that in the presence of domain IV of the *S. aureus* immunomodulator Sbi, disulfide-linked C3d dimers undergo a rare N-terminal 3D domain swapping event, whereby the two C3d molecules in the dimer exchange their N-terminal α1 helices (**Figure 1**). 3D domain swapping has been reported to occur in a diverse range of proteins and is thought to be a mechanism for forming oligomeric states. Although in some cases, the formation of higher-order oligomers can lead to misfolding, aggregation, and disease, there is a subset of proteins whereby domain swapping leads to increased stability and regulates protein function (30). However, no examples of 3D domain swapping have been described in proteins involved in the complement system so far and this is also the first reported example of a ligand-induced domain swap.

The domain swap observed in the C3d^17C^:Sbi-IV dimer complex structurally stabilises the dimer as evidenced by the expansion in interaction surface and enhanced number of intermolecular interactions. In addition, the C3d^17C^ dimer in its Sbi-IV-bound state appears to have a more stable disulfide linkage, evidenced by well-defined electron density for two intact conformers of a C17-C17 interchain disulfide bond at the C3d dimer interface (**Figure 1B** inset). This in contrast to the unliganded C3d^17C^ dimer which only shows a partial disulfide bond (14). Although domain swapped proteins have been reported at acidic pH (3.5-4.0) (31), the helix swap detailed here occurred at neutral pH further supporting its physiological relevance.

**Figure 5:**
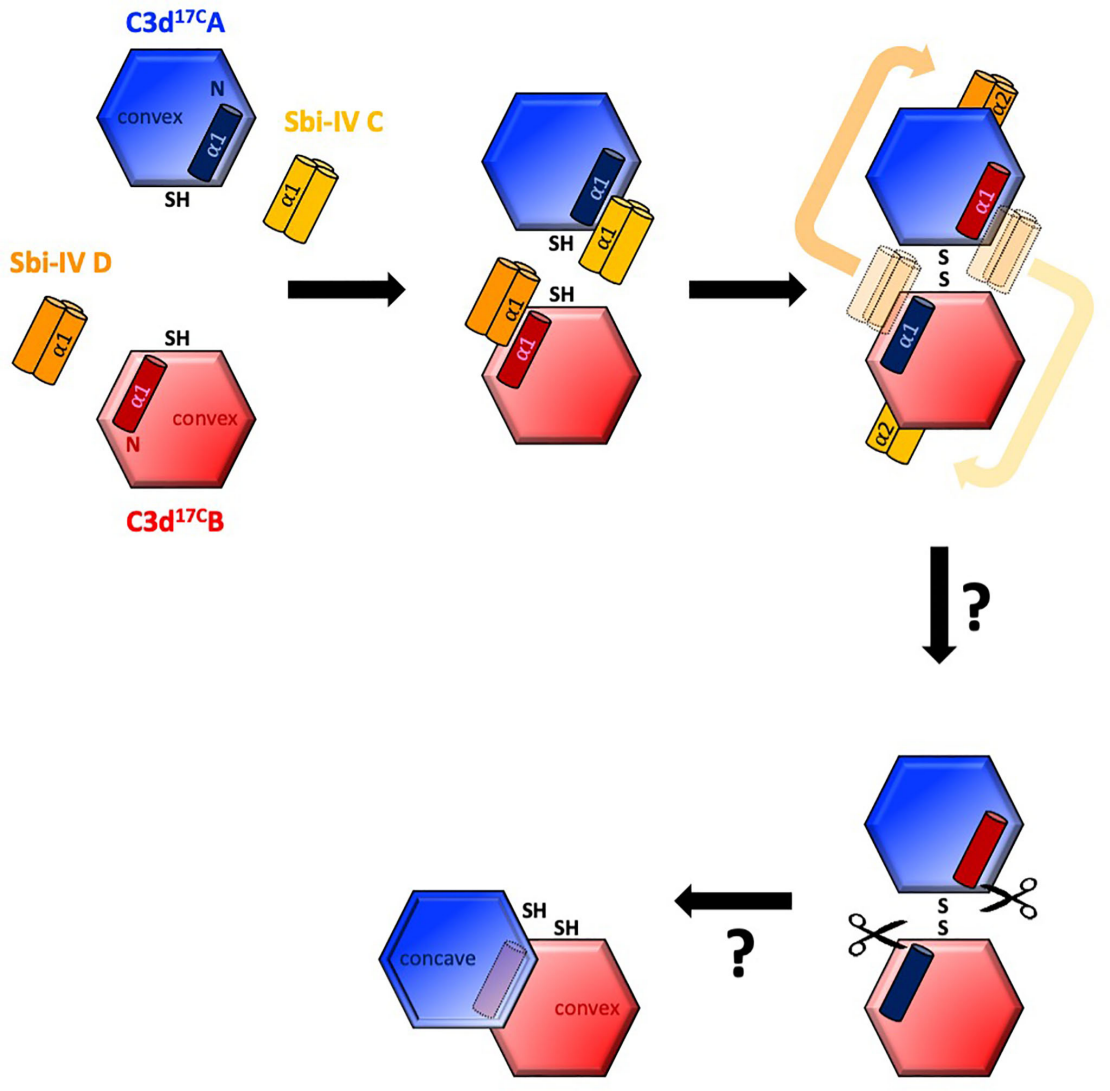
Schematic model depicting the proposed mechanistic basis behind Sbi-IV-induced N-terminal helix swapping of dimeric C3d. **Top left**: In the fluid phase, Sbi-IV helix α1 binds to the N-terminal helix α1 and its adjoining loop on the convex thioester-containing face of C3d^17C^ monomer. **Top middle and right**: When two Sbi-IV-bound C3d^17C^ monomers come into close proximity, Sbi-IV helix α2 is attracted to and binds the concave face of C3d^17C^ (a more favourable higher affinity interaction), pulling C3d^17C^ helix α1 along with it, resulting in a reciprocal exchange of the N-terminal α1 helices of the two C3d^17C^ monomers. **Bottom**: The truncated rat C3dg dimer might be a relic of a helix swapped dimer, in which helix α1 has become more susceptibility to further proteolytic degradation following the 3D domain swap. This leads to the formation of a more compact dimer in which the hydrophobic surfaces exposed after proteolysis of both α1 helices face each other (see also **Supplementary Figure S5**).

Intriguingly, a secondary binding mode for Sbi-IV on the convex surface of C3d revealed previously through X-ray crystallographic, NMR chemical shift perturbation (16) and SAXS analyses (26), is located near the base or hinge region of the swapped C3d^17C^ α1 helix, close to cysteine C17. It is at this position, between helices α1 and α2, where Sbi-IV helix α1 forms interactions with the thioester cysteine C17/1010 and surrounding residues S15/1008 and Q20/1013 of C3d [(**Supplementary Figure S2** (17)]. Thus, this alternative binding mode of Sbi-IV to the hinge region of the N-terminal α1 helix of C3d, which is occluded in the C3d^17C^:Sbi-IV dimer complex by tight dimer interactions, could play an important role in the Sbi-IV-mediated helix swap observed. Particularly, as helix α1 is prone to displacement, which has been shown to occur during the conversion of C3 to C3b (2). The binding of Sbi-IV could further increase the susceptibility of this specific helix in C3d to displacement such that when two C3d monomers bound by Sbi-IV on their convex surfaces are brought into close proximity, the helix α1-bound Sbi-IV molecules migrate to bind the concave face of the opposing C3d molecules, a favoured higher-affinity interaction, causing a reciprocal exchange of the C3d α1 helices followed by disulfide linkage of the thioester cysteine residues at position 17/1010 (**Figure 5**).

It is worth mentioning here that in a previously reported structure of a C3dg dimer, isolated from rat serum (32), the N-termini of both monomers have undergone severe proteolytic truncation. Intriguingly, both monomers lack the exact N-terminal region (M1/994 – G16/1009) (32) that we found to be involved in the helix swap in the C3d^17C^:Sbi-IV dimer complex. The hydrophobic surfaces exposed by the removal of both α1 helices form the dimer-dimer interface in the rat C3dg dimer (**Supplementary Figure S5** and **Figure 5**). Although this dimer is not disulfide linked, its C17 cysteine residues are juxtaposed within ~8Å each other. This could suggest the truncated rat C3dg dimer is a relic of a helix swapped dimer, which became more susceptible to further degradation following the helix α1 swap, thereby leading to the formation of a more compact dimer (**Figure 5** and **Supplementary Figure S5**). In line with this, accessible surface area analysis shows that glycine G16, located N-terminally from the C17-C17 disulfide, is more solvent-exposed in C3d^17C^:Sbi-IV dimer complex, when compared to the unliganded C3d^17C^ dimer, and may therefore be more prone to proteolytic cleavage.

Both the near-UV thermal melt CD analyses as well as the customised tryptophan fluorescence studies presented here (**Figures 2** **–** **4**), show that truncation of Sbi-IV helix α1 abolishes its ability to displace the N-terminal α1 helical region of C3d, providing support for the proposed role of Sbi-IV interactions with the convex binding site of C3d in the molecular mechanism underlying the observed helix swap (**Figure 5**). Our findings also indicate that this helix swap isn’t caused exclusively by the Sbi-IV fragment, but also occurs in the presence of the larger Sbi-III-IV fragment (**Figure 4B**). In addition to establishing that Sbi binding to C3d’s convex surface plays a likely role in the helix swap, we have also determined the significance of individual Sbi-IV residues involved in these interactions. Residues S199 and I204 appear to be important for interactions with the α1 helix hinge region of C3d, whilst the S219 residue located further down the α1 helix of Sbi-IV is likely involved in facilitating binding to the convex surface in the correct orientation and position (**Figure 3**). E201 and K212 residues appear to have a more supportive role in binding, suggesting that once in position they form stabilising interactions with the convex surface (**Figure 3**).

From a broader perspective, Sbi is known to hijack C3b or C3d components in complex with FHR-1 (24), enabling it to upregulate complement activation in the local environment, thus hindering an effective response through depletion of local complement stores in the fluid phase away from the bacterial surface. It has also been shown that these complexes can form dimers *via* the N-terminal domains of FHR-1 (26). The possible incorporation of C3d dimers into these complexes could potentially lead to the formation of even larger multimeric complexes, creating an extensive platform for the upregulation of fluid phase complement. In addition to these complement evasion roles we reveal here that Sbi’s immune-suppressive strategies may even be more intricate, involving the stabilisation of C3d dimers that, as we have shown previously, can crosslink CR2 and selectively modulate B cell activation, possibly to trigger tolerogenic pathways (14) and thereby increase the pathogen’s survivability. Future investigations into hetero-dimerisation of complement components such as C3b or C4b and their potential roles in synergising co-stimulatory molecules *via* CR2-CR2 or CR2-CR3 crosslinking between immune cells, could provide even further insights into the complex links between various aspects of our immune system.

In summary, our novel discovery of Sbi domain IV’s ability to induce a unique structurally stabilising domain swap in dimeric C3d reveals another dimension in the complex relationship between *S. aureus* and the immune system. Overall, our findings shed light on a fundamental aspect of complement and have the potential to inform the design of novel therapeutics for autoimmune diseases and vaccine design in the future.

## Materials and Methods

### Expression and Purification of Recombinant Proteins

The DNA sequences of human C3d (residues 1-310) with a cysteine to alanine substitution at position 17 (C3d^17A^) was previously cloned into the pET15b expression plasmid (33). To reproduce the wild-type sequence, the alanine at position 17 of the C3d^17A^ construct was reverted to a cysteine (C3d^17C^) using site-directed mutagenesis (14). Single amino acid mutants of C3d^17C^ with an alanine to histidine substitution at position 4(C3d)/997(pre-pro C3) (C3d^A4H^) or with a tryptophan to alanine substitution at position 41(C3d)/1034(pre-pro C3) (C3d^W41A^) were generated through site directed mutagenesis following the protocol detailed in the QuikChange II site-directed mutagenesis kit (Agilent) using Pfu polymerase (Promega) and the primers detailed in **Supplementary Table S3**.

All C3d constructs were expressed in the *Escherichia coli* Shuffle T7 cells (NEB) and purified using cation exchange chromatography (column: 1 mL HiTrap SP Sepharose HP [Cytivia], binding buffer: 50 mM MES, pH 5.5, elution buffer: 50 mM MES, 500 mM NaCl, pH 5.5) followed by size exclusion chromatography (column: HiLoad 16/600 Superdex 200 prep grade [GE Healthcare], buffer: 20 mM Tris, 150 mM NaCl, pH 7.4).

The DNA sequences of the Sbi constructs composed of domain IV (150-266 of full-length Sbi) and domains III and IV (198-266 of full-length Sbi), both bearing N-terminal 6x His-tags were previously cloned into the pQE-30 Xa plasmid [Sbi-IV (14); Sbi-III-IV (16)]. The DNA sequence of a truncated Sbi-IV construct lacking 11 residues located at the N-terminus of helix α1 (198-208 of full-length Sbi) (Sbi-IV^V198-D208del^) was produced using PCR with designed primers (**Supplementary Table S3**) and cloned into the pET-28a plasmid. Various single amino acid mutants of Sbi-IV where residues at positions 199, 201, 204, 208, 212, 219 and 243 were substituted to alanines were produced through site-directed mutagenesis as described above for C3d using primers detailed in **Supplementary Table S3**.

All Sbi constructs were expressed in *E. coli* BL21(DE3) (Sigma Aldrich) cells and purified using nickel-affinity (column: 1mL HisTrap FF [Cytivia], binding buffer: 50 mM Tris, 15 mM NaCl, 20 mM Imidazole, pH 7.4, elution buffer: 50 mM Tris, 150 mM NaCl, 500 mM Imidazole, pH 7.4) and size exclusion chromatography (details as described above for C3d).

### Crystallisation, Data Collection and Structure Determination

Crystallisation was performed at 18°C using the hanging drop vapour diffusion method. For the C3d^17C^ dimer-Sbi-IV complex structure, needle-like co-crystals at a 1:1 molar (288 µM) ratio of Sbi-IV (2.66 mg ml^-1^) and C3d^17C^ (10 mg ml^-1^) appeared in the condition containing 0.2 M Sodium citrate tribasic dihydrate, 20% PEG 3350 (PACT *premier* condition E11, Molecular Dimensions) (measured pH: 8.07).

Crystals were mounted on loops, flash-frozen in liquid nitrogen and X-ray diffraction data collected on the I04 beamline Dectris PILATUS 6M pixel detector at the Diamond Light Source synchrotron (Oxfordshire, UK) (See **Supplementary Table S1** for data collection statistics). Integration of diffraction images and data reduction were performed using Xia2-DIALS and AIMLESS respectively (C3d^17C^ dimer-Sbi-IV complex structure: resolution: 2.4 Å, images 1601-3600 excluded). The automated BALBES pipeline and COOT were used for molecular replacement and model building. Refinement was carried out in REFMAC and Phenix (refinement statistics can be found in **Supplementary Table S2**) and UCSF Chimera was used for superpositioning and generation of images. Bond numbers and buried surface area were calculated with PDBePISA. The structure has been submitted to the Protein Data Bank with PDB submission code 6RMU (C3d^17C^ dimer-Sbi-IV complex).

### Circular Dichroism (CD) Spectroscopy

For tertiary structure thermal melt analyses, proteins were buffer exchanged into 10mM sodium phosphate pH 7.4 using Zeba™ Spin desalting columns (ThermoFisher) according to the manufacturer’s instructions. Buffer-corrected CD measurements of 87.5 µM C3d^17C^ or C3d^17A^ alone or in the presence of an equimolar concentration of wild-type Sbi-IV, Sbi-IV^V198-D208del^ and Sbi-IV mutants were acquired at wavelengths between 250 and 320 nm in 1 nm increments, and at temperatures ranging from 5°C to 85°C in 5°C increments followed by a final reading at 20°C. All measurements were obtained on a Chirascan spectrometer (Applied Photophysics) with a bandwidth of 2 nm and 2 second time per point. Millidegree values were converted to units of mean residue ellipticity, and deconvolution of secondary structural data was performed using DichroWeb. Melting temperature values were estimated using sigmoidal fits of thermal denaturation curves at different wavelengths performed using the Boltzmann function in GraphPad Prism (version 9.3.1). See **Figures 2A, B** respectively for C3d^17C^ and C3d^17A^ sigmoidal fits of thermal denaturation curves used for T_m_ calculations.

### C3d Heterodimer Formation

The C3d^A4H^ and C3d^W41A^ proteins were initially buffer exchanged into 10 mM sodium phosphate pH 7.4 as detailed above. Prior to fluorometric analysis, the two mutant proteins at a concentration of 1 mg mL^-1^ were mixed together and treated with tris (2-carboxyethyl) phosphine (TCEP) (Merck) in TNE buffer (1 M Tris, 150 mM NaCl, 5 mM EDTA, pH 7.5) for 1 hour at 4°C, to partially reduce the proteins and cause any homodimers to dissociate. TCEP was subsequently removed allowing the formation of heterodimers by dialysis overnight at 4°C into 10 mM sodium phosphate pH 7.4 using Slide-A-Lyzer™ dialysis cassettes (ThermoFisher).

### Fluorescence Spectroscopy

Following dialysis the C3d^A4H^/C3d^W41A^ protein solution was concentrated and used for fluorometry experiments. Samples containing, 100 µM of the C3d^A4H^/C3d^W41A^ solution alone or in the presence of an equimolar concentration of Sbi-IV, Sbi-III-IV and Sbi-IV^V198-D208del^ were excited at the 295 nm wavelength and emissions between the 300 and 450 nm wavelengths were recorded. All measurements were obtained on an LS50B Luminescence Spectrometer (PerkinElmer) at room temperature using 6nm slits and a 250 ms scan speed. For analysis, the emission spectra were visualised in GraphPad Prism (version 9.3.1) as XY scatter representations. The Sbi construct intensity change was calculated for individual emission spectra by comparing the intensity reading for the C3d^A4H^/C3d^W41A^ peak alone with the Sbi intensity reading at the corresponding wavelength. These intensity changes were calculated as percentages and visualised as column representations that displayed the individual points, as well as the mean and standard deviation.

## Data Availability Statement

The original contributions presented in the study are included in the article/**Supplementary Material**. Further inquiries can be directed to the corresponding author/s.

## Author Contributions

JE, CD, AGW, AAW, and RD designed the experiments. CB performed preliminary structural studies and AAW performed the crystallisation. SC and AAW reprocessed the crystallography data and refined the structures. RD produced the truncated and mutated constructs. AAW and RD performed the circular dichroism experiments. RD performed the TCEP reduction, supported by RM. RD performed the tryptophan fluorescence spectroscopy experiments. JE and RD wrote the manuscript with valuable contributions from all the authors. All authors contributed to the article and approved the submitted version.

## Funding

This research was supported by the Biotechnology and Biological Sciences Research Council Follow On Fund BB/N022165/1. AAW was sponsored by a PhD studentship granted by Raoul and Catherine Hughes and the University of Bath Alumni Fund. RD was supported by a Medical Research Council GW4 Doctoral Training Partnership (MR/N0137941/1).

## Conflict of Interest

The authors declare that the research was conducted in the absence of any commercial or financial relationships that could be construed as a potential conflict of interest.

## Publisher’s Note

All claims expressed in this article are solely those of the authors and do not necessarily represent those of their affiliated organizations, or those of the publisher, the editors and the reviewers. Any product that may be evaluated in this article, or claim that may be made by its manufacturer, is not guaranteed or endorsed by the publisher.
