# Supplementary material for: Staphylococcal Complement Evasion Protein Sbi Stabilises C3d Dimers by Inducing an N-Terminal Helix Swap
Source: Front Immunol. 2022 May 25;13:892234. doi: 10.3389/fimmu.2022.892234 (PMC9174452; doi:10.3389/fimmu.2022.892234)
Supplement: Supplementary file 1 [file Presentation_1.pptx]

## Slide 1
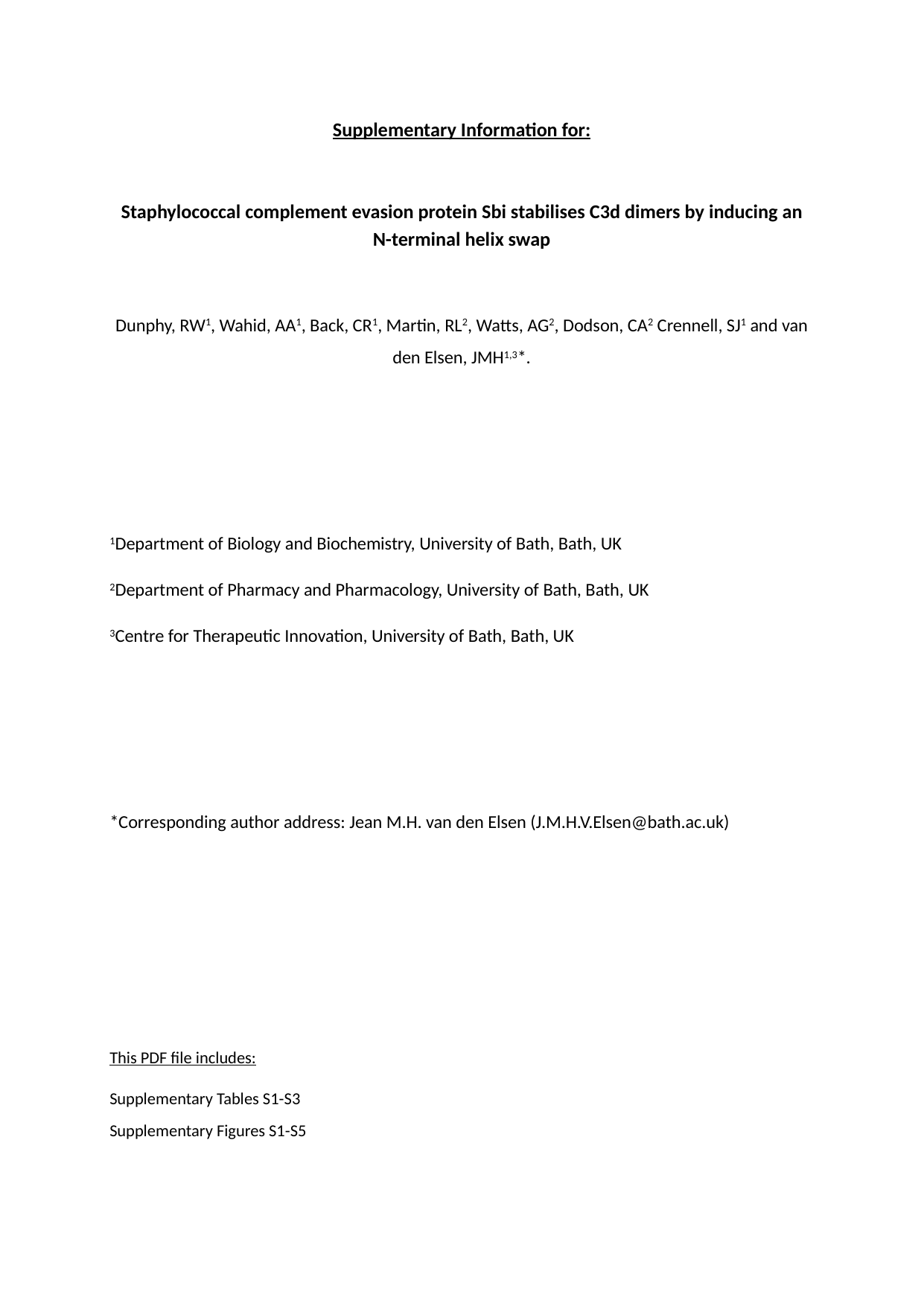

Supplementary Information for:
Staphylococcal complement evasion protein Sbi stabilises C3d dimers by inducing an N-terminal helix swap
Dunphy, RW1, Wahid, AA1, Back, CR1, Martin, RL2, Watts, AG2, Dodson, CA2 Crennell, SJ1 and van den Elsen, JMH1,3*.
1Department of Biology and Biochemistry, University of Bath, Bath, UK
2Department of Pharmacy and Pharmacology, University of Bath, Bath, UK
3Centre for Therapeutic Innovation, University of Bath, Bath, UK
*Corresponding author address: Jean M.H. van den Elsen (J.M.H.V.Elsen@bath.ac.uk)
This PDF file includes:
Supplementary Tables S1-S3
Supplementary Figures S1-S5

## Slide 2
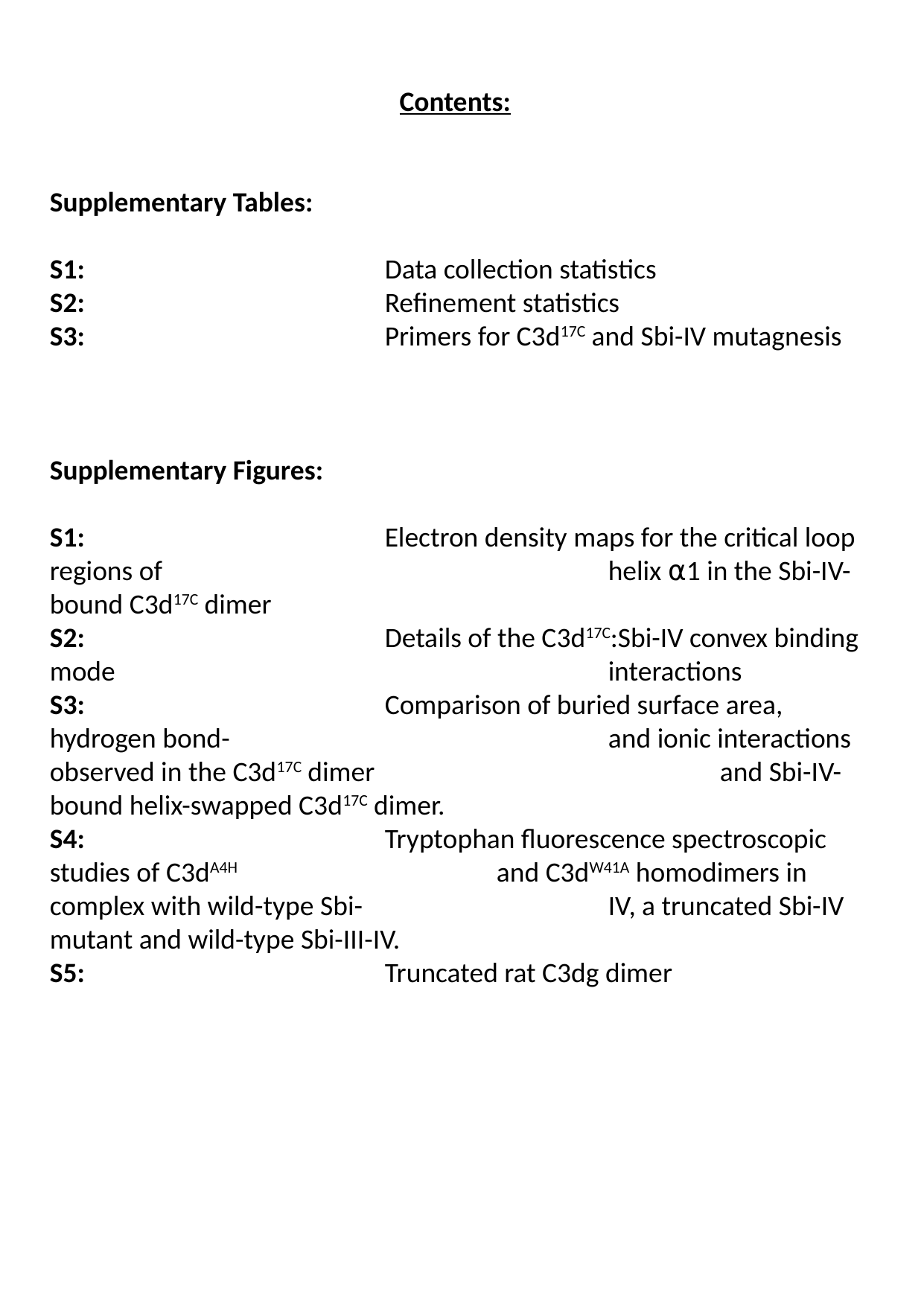

Contents:
Supplementary Tables:
S1: 			Data collection statistics
S2: 			Refinement statistics
S3: 			Primers for C3d17C and Sbi-IV mutagnesis
Supplementary Figures:
S1:			Electron density maps for the critical loop regions of 				helix ⍺1 in the Sbi-IV-bound C3d17C dimer
S2: 			Details of the C3d17C:Sbi-IV convex binding mode 					interactions
S3:		 	Comparison of buried surface area, hydrogen bond- 				and ionic interactions observed in the C3d17C dimer 				and Sbi-IV-bound helix-swapped C3d17C dimer.
S4: 			Tryptophan fluorescence spectroscopic studies of C3dA4H 			and C3dW41A homodimers in complex with wild-type Sbi-			IV, a truncated Sbi-IV mutant and wild-type Sbi-III-IV.
S5:			Truncated rat C3dg dimer

## Slide 3
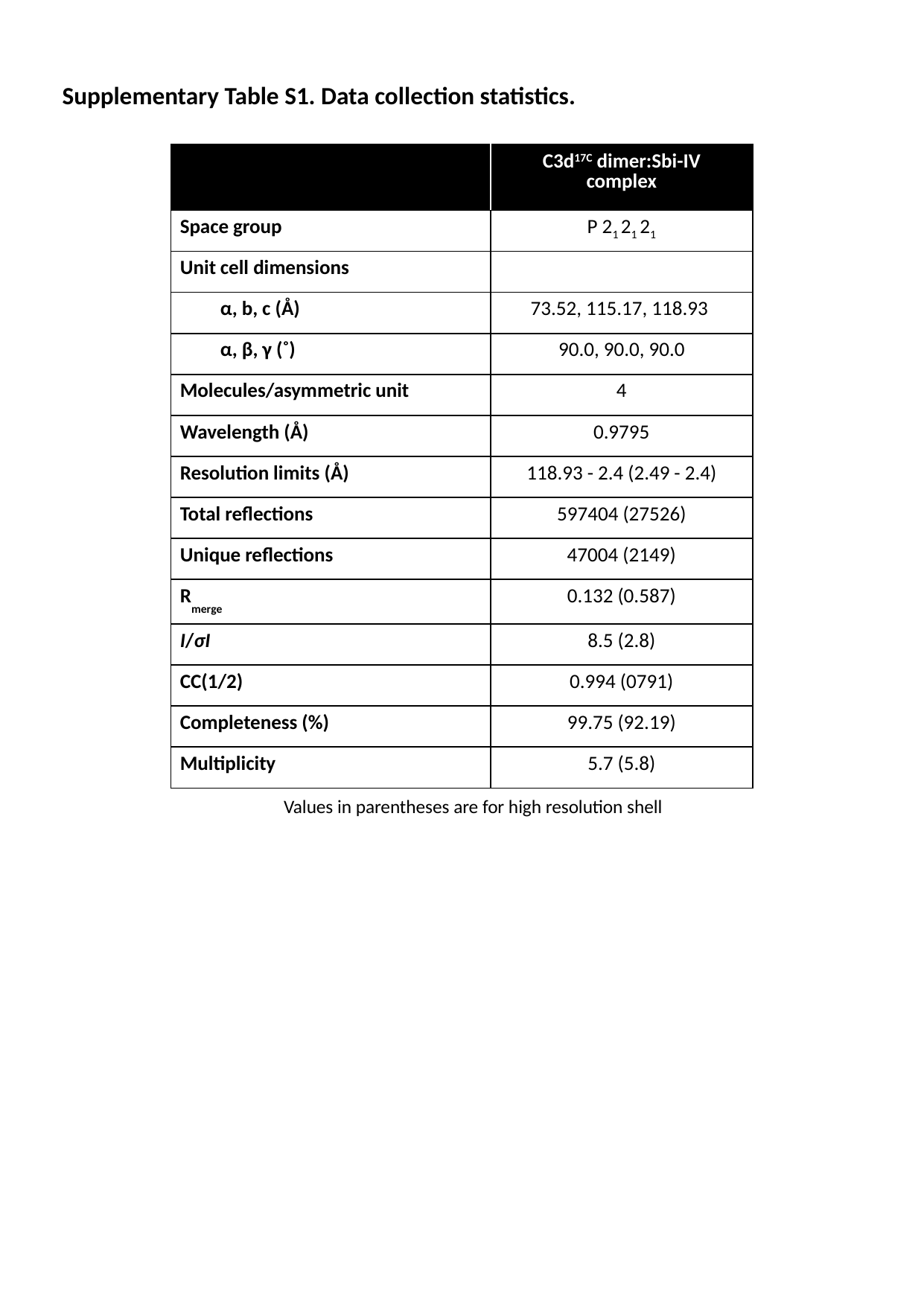

Supplementary Table S1. Data collection statistics.
| | C3d17C dimer:Sbi-IV complex |
| --- | --- |
| Space group | P 21 21 21 |
| Unit cell dimensions | |
| α, b, c (Å) | 73.52, 115.17, 118.93 |
| α, β, γ (˚) | 90.0, 90.0, 90.0 |
| Molecules/asymmetric unit | 4 |
| Wavelength (Å) | 0.9795 |
| Resolution limits (Å) | 118.93 - 2.4 (2.49 - 2.4) |
| Total reflections | 597404 (27526) |
| Unique reflections | 47004 (2149) |
| Rmerge | 0.132 (0.587) |
| I/σI | 8.5 (2.8) |
| CC(1/2) | 0.994 (0791) |
| Completeness (%) | 99.75 (92.19) |
| Multiplicity | 5.7 (5.8) |
Values in parentheses are for high resolution shell

## Slide 4
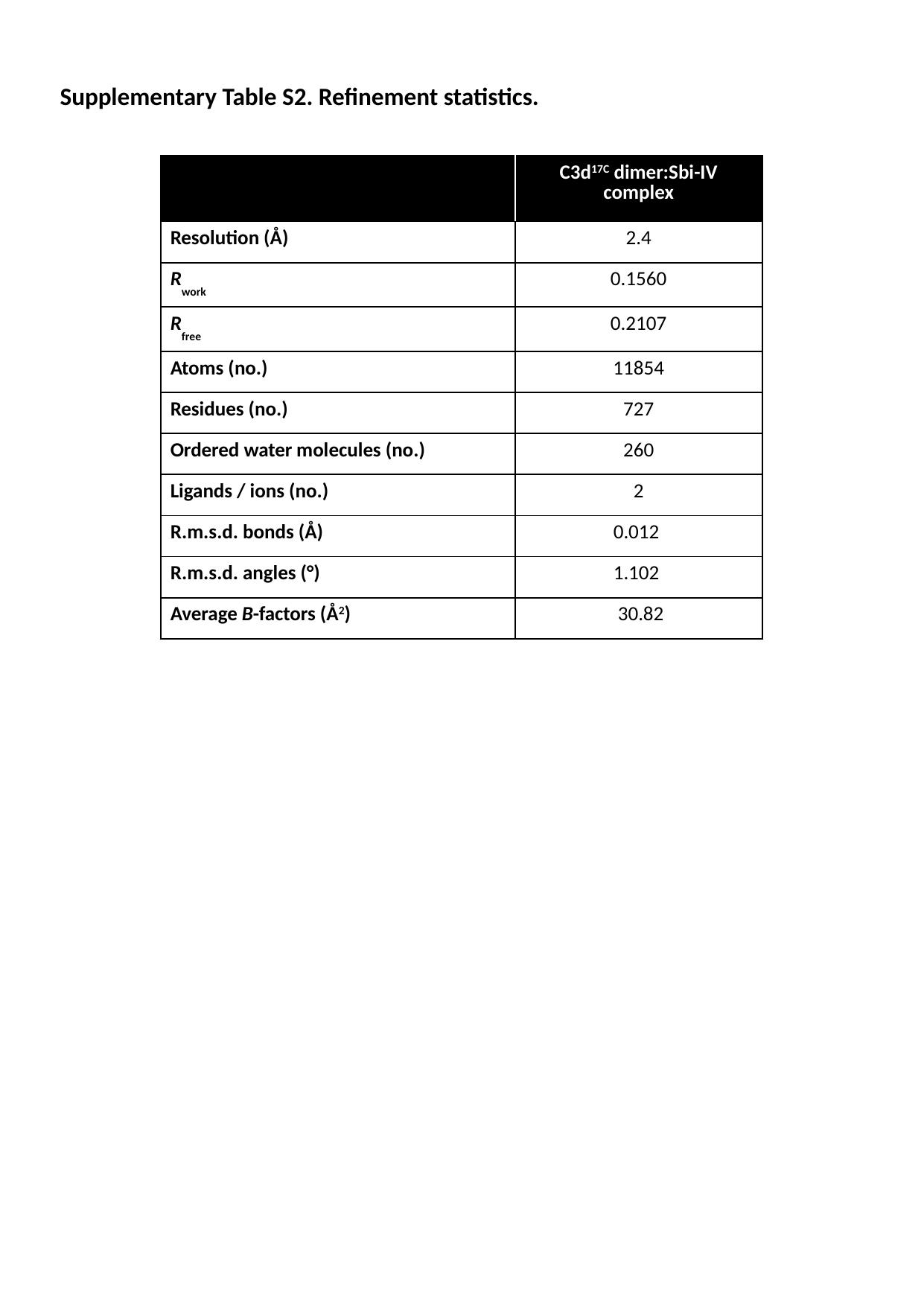

Supplementary Table S2. Refinement statistics.
| | C3d17C dimer:Sbi-IV complex |
| --- | --- |
| Resolution (Å) | 2.4 |
| Rwork | 0.1560 |
| Rfree | 0.2107 |
| Atoms (no.) | 11854 |
| Residues (no.) | 727 |
| Ordered water molecules (no.) | 260 |
| Ligands / ions (no.) | 2 |
| R.m.s.d. bonds (Å) | 0.012 |
| R.m.s.d. angles (°) | 1.102 |
| Average B-factors (Å2) | 30.82 |

## Slide 5
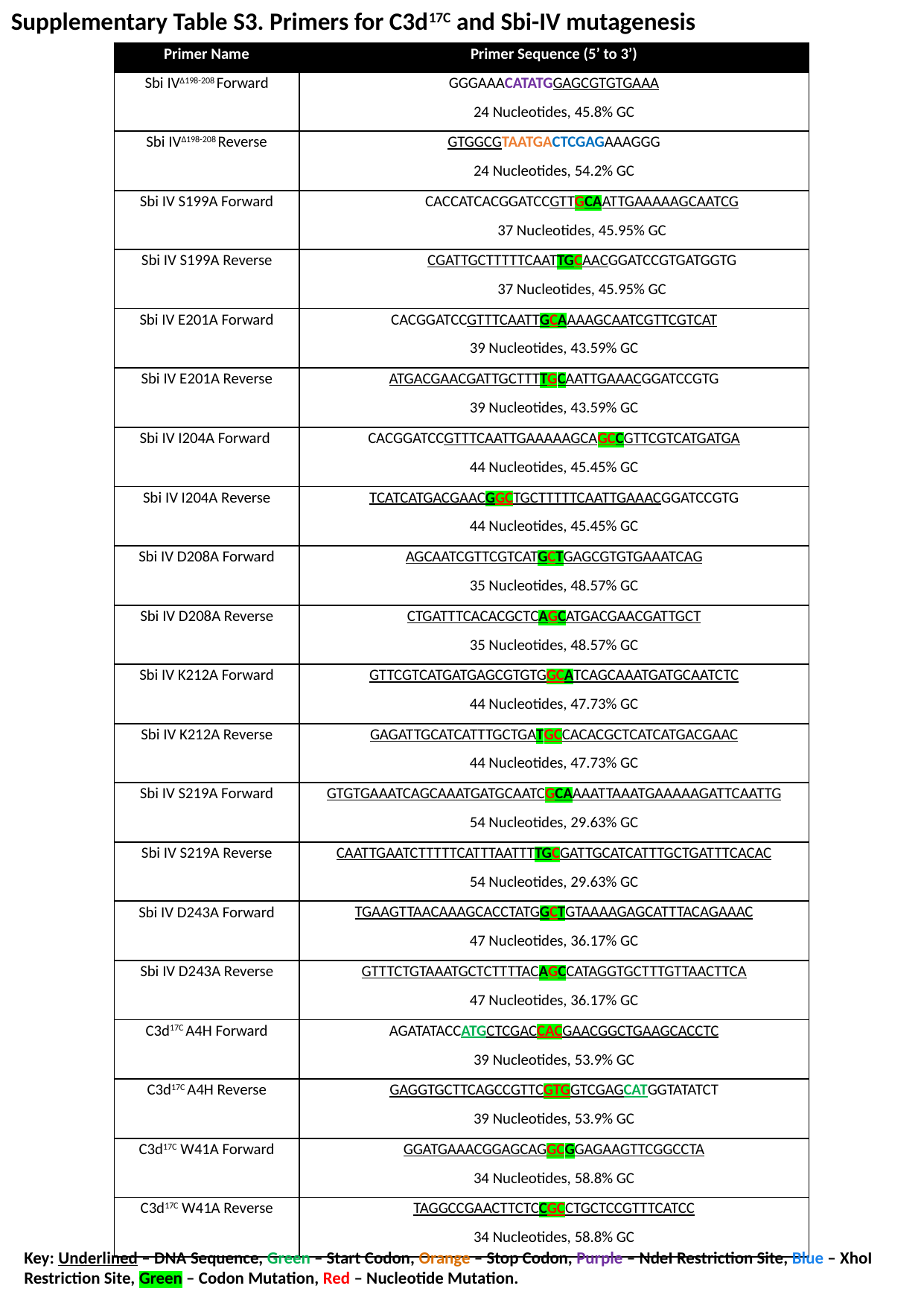

Supplementary Table S3. Primers for C3d17C and Sbi-IV mutagenesis
| Primer Name | Primer Sequence (5’ to 3’) |
| --- | --- |
| Sbi IVΔ198-208 Forward | GGGAAACATATGGAGCGTGTGAAA 24 Nucleotides, 45.8% GC |
| Sbi IVΔ198-208 Reverse | GTGGCGTAATGACTCGAGAAAGGG 24 Nucleotides, 54.2% GC |
| Sbi IV S199A Forward | CACCATCACGGATCCGTTGCAATTGAAAAAGCAATCG 37 Nucleotides, 45.95% GC |
| Sbi IV S199A Reverse | CGATTGCTTTTTCAATTGCAACGGATCCGTGATGGTG 37 Nucleotides, 45.95% GC |
| Sbi IV E201A Forward | CACGGATCCGTTTCAATTGCAAAAGCAATCGTTCGTCAT 39 Nucleotides, 43.59% GC |
| Sbi IV E201A Reverse | ATGACGAACGATTGCTTTTGCAATTGAAACGGATCCGTG 39 Nucleotides, 43.59% GC |
| Sbi IV I204A Forward | CACGGATCCGTTTCAATTGAAAAAGCAGCCGTTCGTCATGATGA 44 Nucleotides, 45.45% GC |
| Sbi IV I204A Reverse | TCATCATGACGAACGGCTGCTTTTTCAATTGAAACGGATCCGTG 44 Nucleotides, 45.45% GC |
| Sbi IV D208A Forward | AGCAATCGTTCGTCATGCTGAGCGTGTGAAATCAG 35 Nucleotides, 48.57% GC |
| Sbi IV D208A Reverse | CTGATTTCACACGCTCAGCATGACGAACGATTGCT 35 Nucleotides, 48.57% GC |
| Sbi IV K212A Forward | GTTCGTCATGATGAGCGTGTGGCATCAGCAAATGATGCAATCTC 44 Nucleotides, 47.73% GC |
| Sbi IV K212A Reverse | GAGATTGCATCATTTGCTGATGCCACACGCTCATCATGACGAAC 44 Nucleotides, 47.73% GC |
| Sbi IV S219A Forward | GTGTGAAATCAGCAAATGATGCAATCGCAAAATTAAATGAAAAAGATTCAATTG 54 Nucleotides, 29.63% GC |
| Sbi IV S219A Reverse | CAATTGAATCTTTTTCATTTAATTTTGCGATTGCATCATTTGCTGATTTCACAC 54 Nucleotides, 29.63% GC |
| Sbi IV D243A Forward | TGAAGTTAACAAAGCACCTATGGCTGTAAAAGAGCATTTACAGAAAC 47 Nucleotides, 36.17% GC |
| Sbi IV D243A Reverse | GTTTCTGTAAATGCTCTTTTACAGCCATAGGTGCTTTGTTAACTTCA 47 Nucleotides, 36.17% GC |
| C3d17C A4H Forward | AGATATACCATGCTCGACCACGAACGGCTGAAGCACCTC 39 Nucleotides, 53.9% GC |
| C3d17C A4H Reverse | GAGGTGCTTCAGCCGTTCGTGGTCGAGCATGGTATATCT 39 Nucleotides, 53.9% GC |
| C3d17C W41A Forward | GGATGAAACGGAGCAGGCGGAGAAGTTCGGCCTA 34 Nucleotides, 58.8% GC |
| C3d17C W41A Reverse | TAGGCCGAACTTCTCCGCCTGCTCCGTTTCATCC 34 Nucleotides, 58.8% GC |
Key: Underlined – DNA Sequence, Green – Start Codon, Orange – Stop Codon, Purple – NdeI Restriction Site, Blue – XhoI Restriction Site, Green – Codon Mutation, Red – Nucleotide Mutation.

## Slide 6
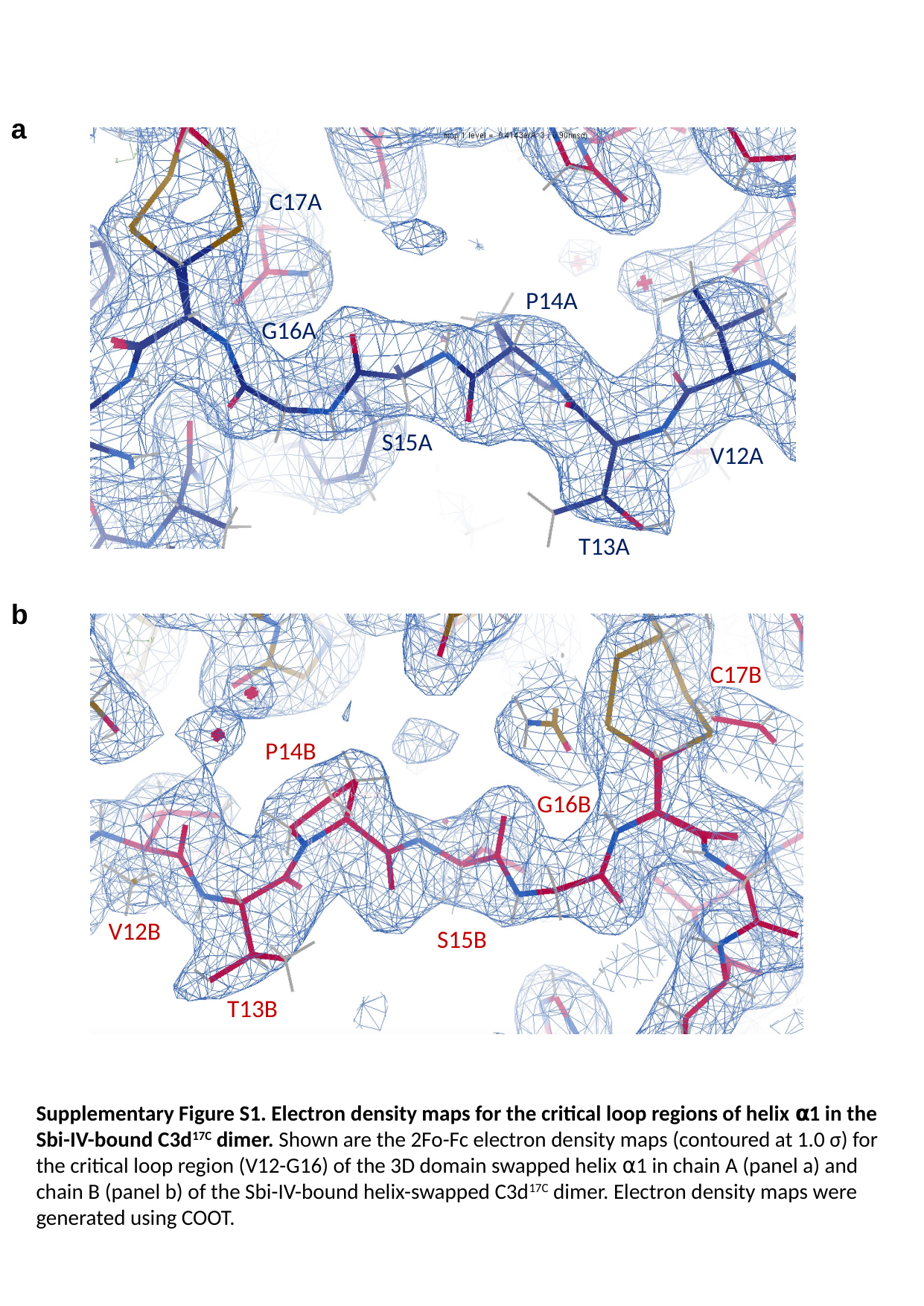

a
C17A
P14A
G16A
S15A
V12A
T13A
b
C17B
P14B
G16B
V12B
S15B
T13B
Supplementary Figure S1. Electron density maps for the critical loop regions of helix ⍺1 in the Sbi-IV-bound C3d17C dimer. Shown are the 2Fo-Fc electron density maps (contoured at 1.0 σ) for the critical loop region (V12-G16) of the 3D domain swapped helix ⍺1 in chain A (panel a) and chain B (panel b) of the Sbi-IV-bound helix-swapped C3d17C dimer. Electron density maps were generated using COOT.

## Slide 7
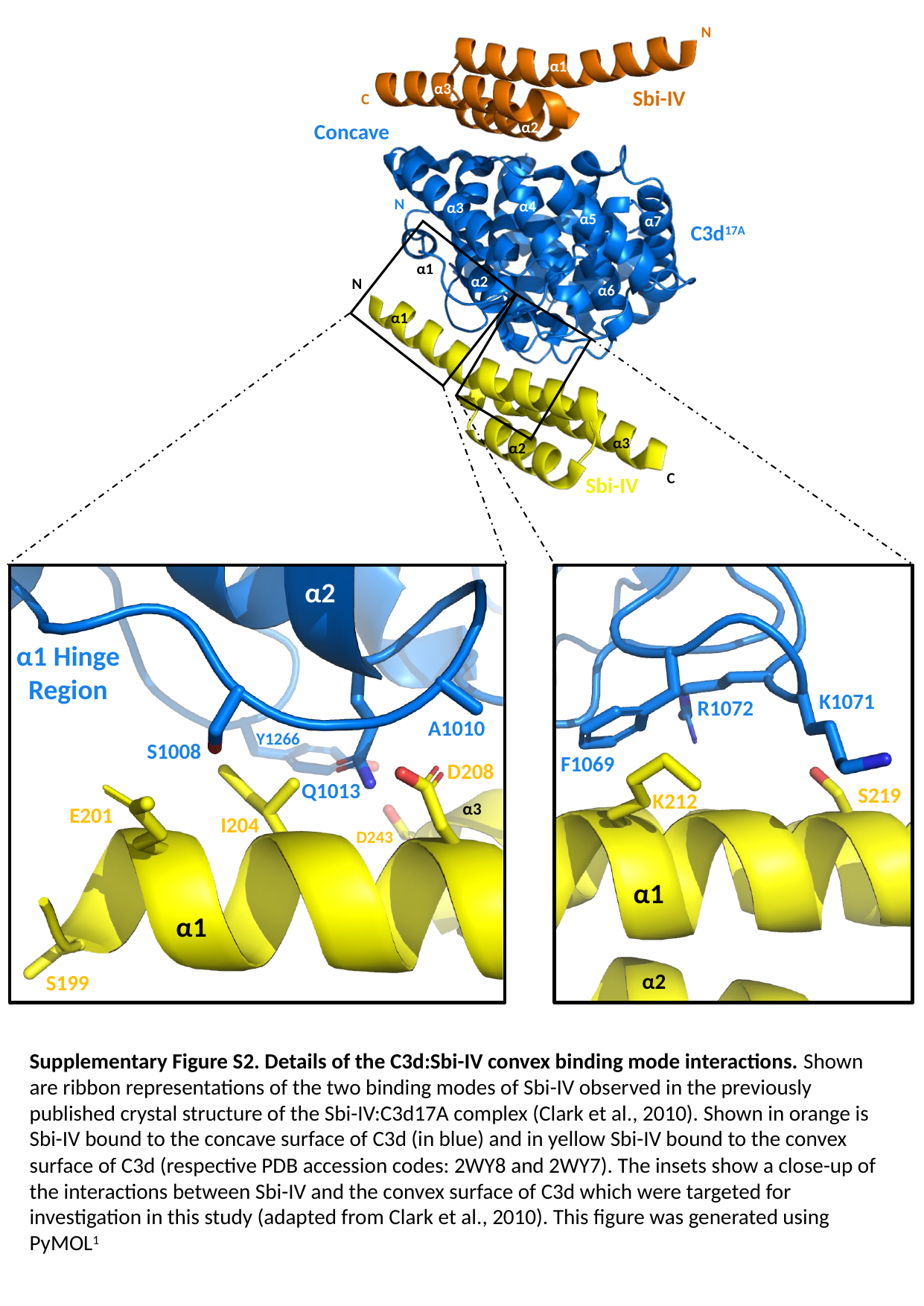

N
α1
α3
Sbi-IV
C
α2
Concave
N
α4
α3
α5
α7
C3d17A
α1
α2
N
α6
α1
α3
α2
C
Sbi-IV
α2
α1 Hinge Region
K1071
R1072
A1010
Y1266
S1008
F1069
D208
Q1013
S219
K212
α3
E201
I204
D243
α1
α1
α2
S199
Supplementary Figure S2. Details of the C3d:Sbi-IV convex binding mode interactions. Shown are ribbon representations of the two binding modes of Sbi-IV observed in the previously published crystal structure of the Sbi-IV:C3d17A complex (Clark et al., 2010). Shown in orange is Sbi-IV bound to the concave surface of C3d (in blue) and in yellow Sbi-IV bound to the convex surface of C3d (respective PDB accession codes: 2WY8 and 2WY7). The insets show a close-up of the interactions between Sbi-IV and the convex surface of C3d which were targeted for investigation in this study (adapted from Clark et al., 2010). This figure was generated using PyMOL1

## Slide 8
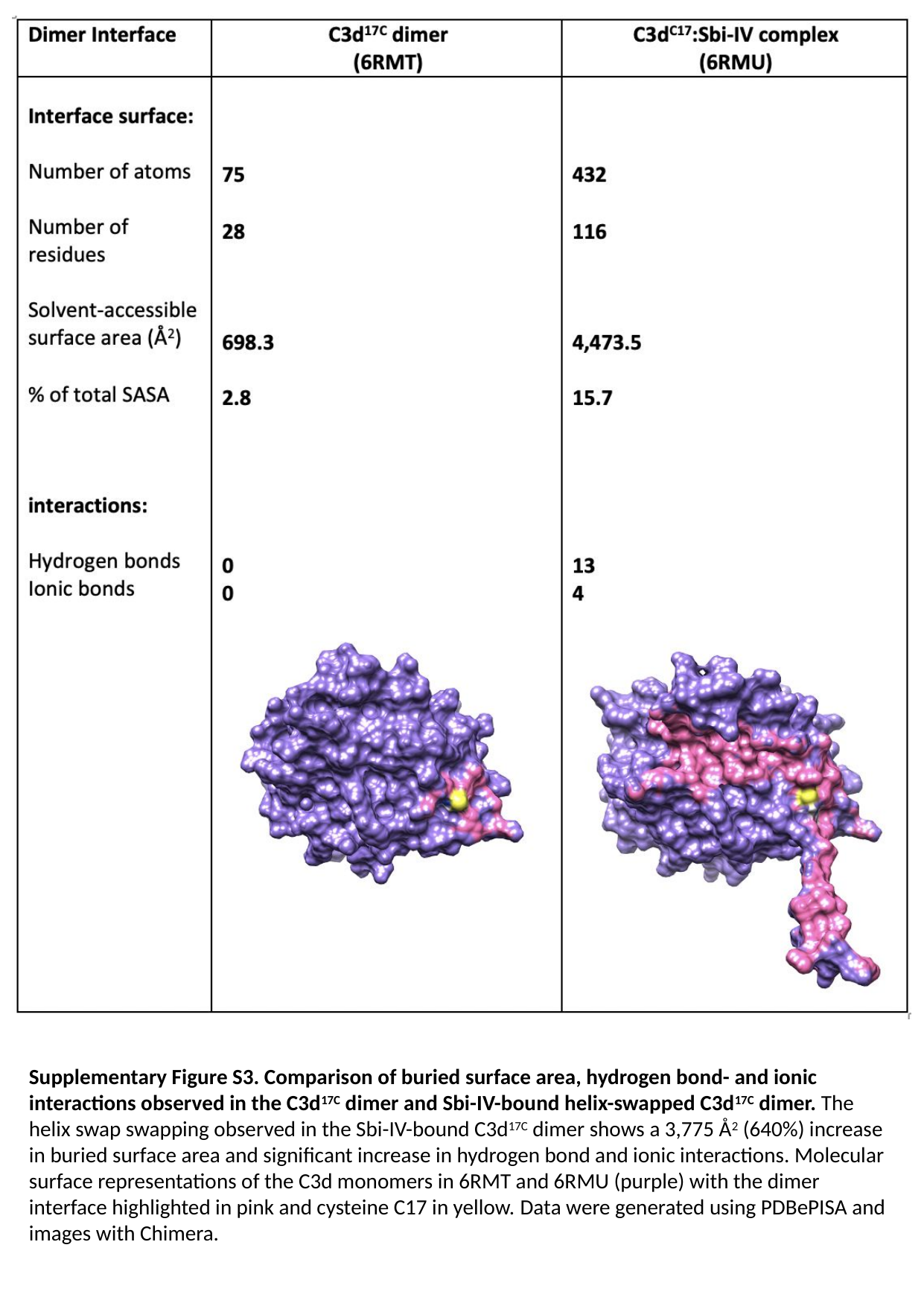

Supplementary Figure S3. Comparison of buried surface area, hydrogen bond- and ionic interactions observed in the C3d17C dimer and Sbi-IV-bound helix-swapped C3d17C dimer. The helix swap swapping observed in the Sbi-IV-bound C3d17C dimer shows a 3,775 Å2 (640%) increase in buried surface area and significant increase in hydrogen bond and ionic interactions. Molecular surface representations of the C3d monomers in 6RMT and 6RMU (purple) with the dimer interface highlighted in pink and cysteine C17 in yellow. Data were generated using PDBePISA and images with Chimera.

## Slide 9
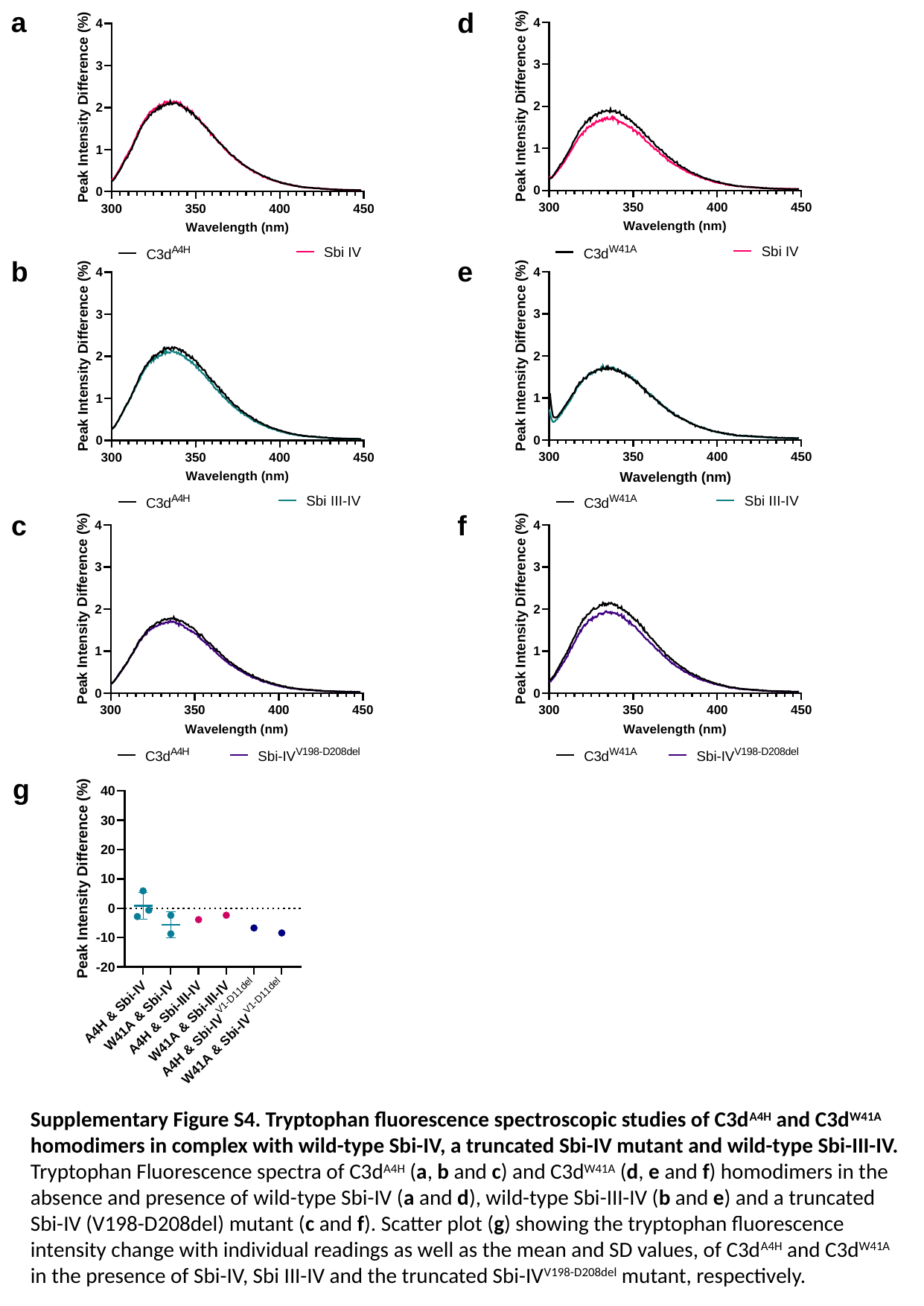

d
a
b
e
c
f
g
Supplementary Figure S4. Tryptophan fluorescence spectroscopic studies of C3dA4H and C3dW41A homodimers in complex with wild-type Sbi-IV, a truncated Sbi-IV mutant and wild-type Sbi-III-IV. Tryptophan Fluorescence spectra of C3dA4H (a, b and c) and C3dW41A (d, e and f) homodimers in the absence and presence of wild-type Sbi-IV (a and d), wild-type Sbi-III-IV (b and e) and a truncated Sbi-IV (V198-D208del) mutant (c and f). Scatter plot (g) showing the tryptophan fluorescence intensity change with individual readings as well as the mean and SD values, of C3dA4H and C3dW41A in the presence of Sbi-IV, Sbi III-IV and the truncated Sbi-IVV198-D208del mutant, respectively.

## Slide 10
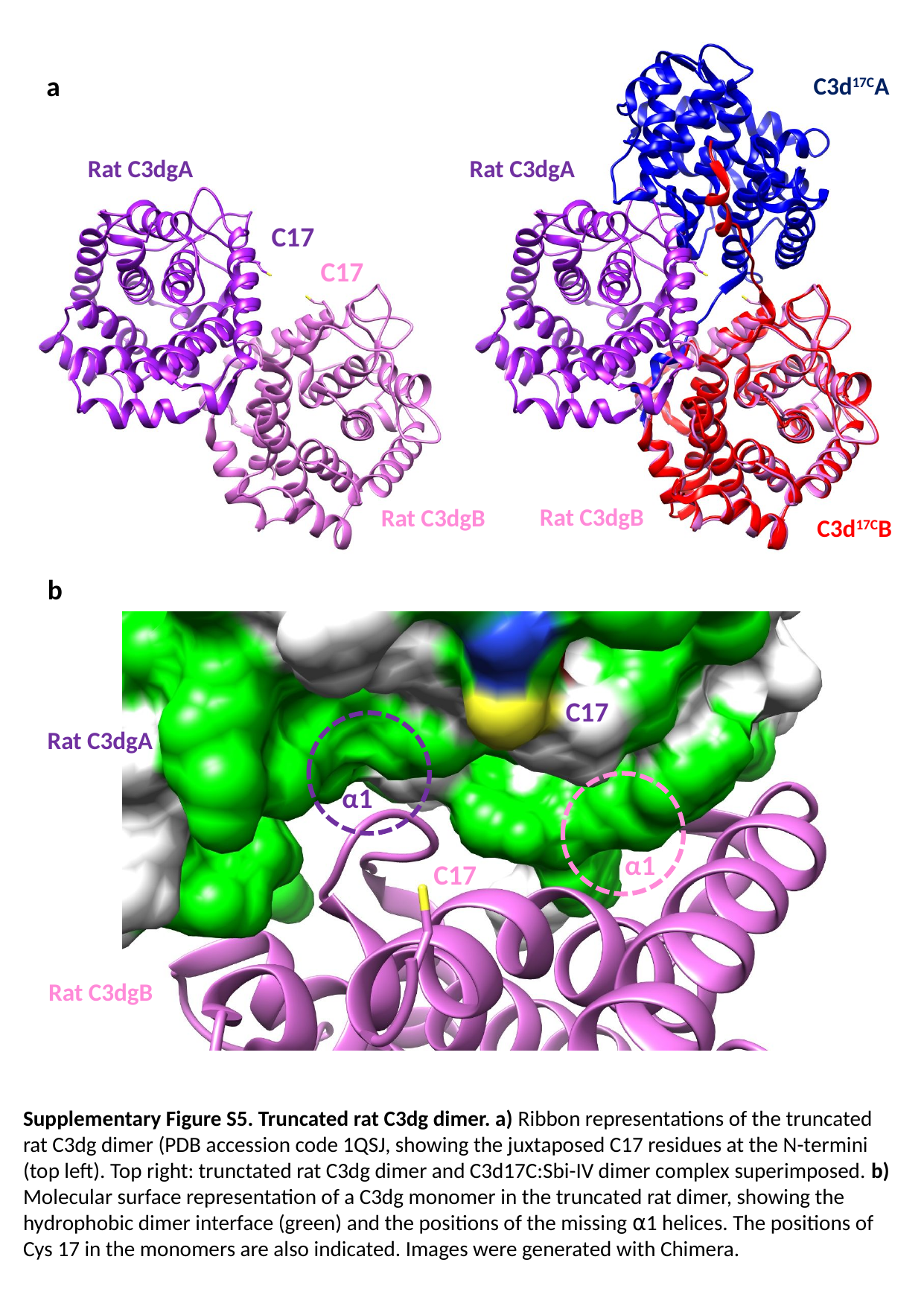

a
C3d17CA
Rat C3dgA
Rat C3dgA
C17
C17
Rat C3dgB
Rat C3dgB
C3d17CB
b
C17
Rat C3dgA
α1
α1
C17
Rat C3dgB
Supplementary Figure S5. Truncated rat C3dg dimer. a) Ribbon representations of the truncated rat C3dg dimer (PDB accession code 1QSJ, showing the juxtaposed C17 residues at the N-termini (top left). Top right: trunctated rat C3dg dimer and C3d17C:Sbi-IV dimer complex superimposed. b) Molecular surface representation of a C3dg monomer in the truncated rat dimer, showing the hydrophobic dimer interface (green) and the positions of the missing ⍺1 helices. The positions of Cys 17 in the monomers are also indicated. Images were generated with Chimera.

## Slide 11
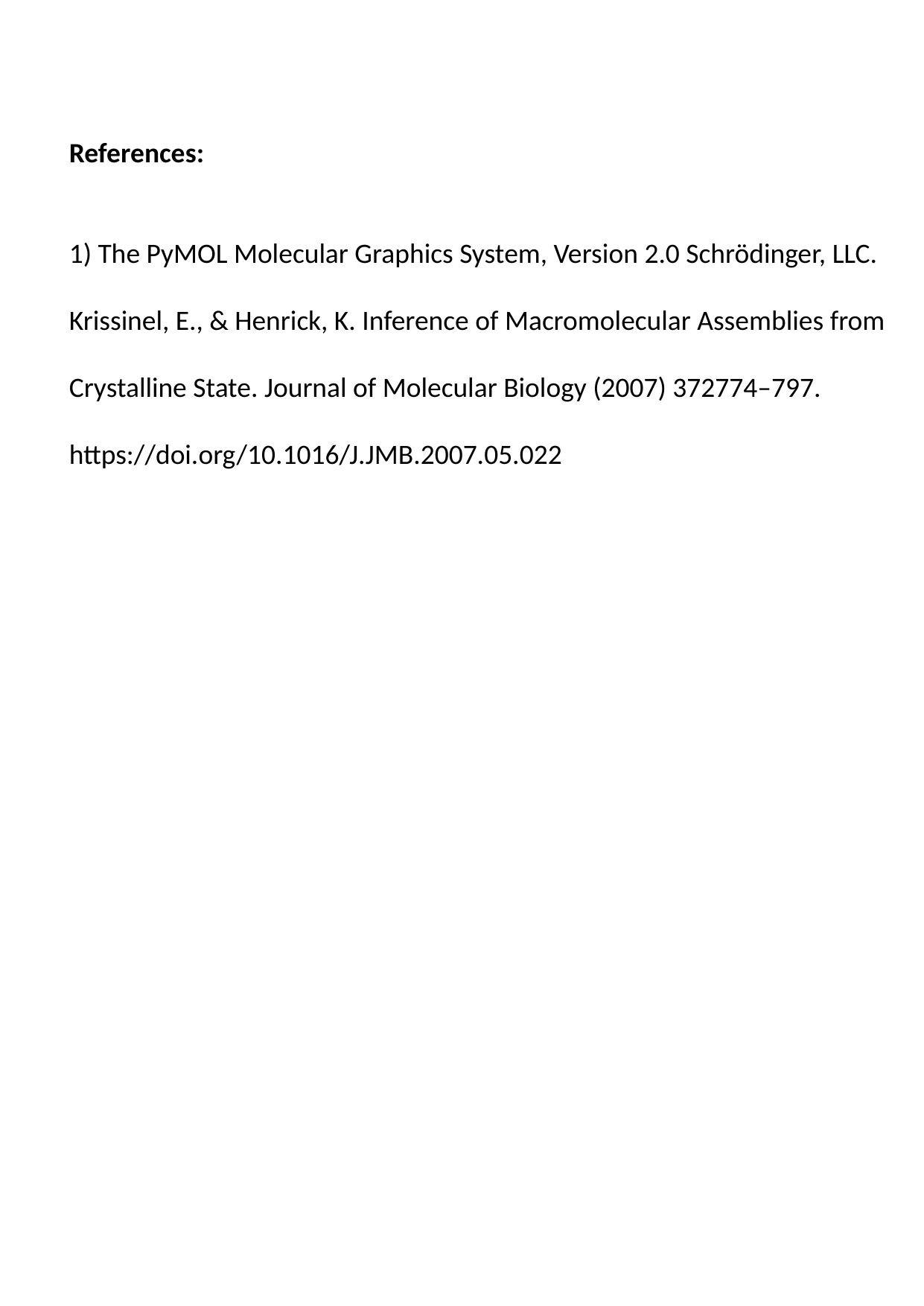

References:
1) The PyMOL Molecular Graphics System, Version 2.0 Schrödinger, LLC.
Krissinel, E., & Henrick, K. Inference of Macromolecular Assemblies from
Crystalline State. Journal of Molecular Biology (2007) 372774–797.
https://doi.org/10.1016/J.JMB.2007.05.022
